# Microplastics Exposure Impact on Lung Cancer—Literature Review

**DOI:** 10.3390/cancers17223616

**Published:** 2025-11-10

**Authors:** Grzegorz Sychowski, Hanna Romanowicz, Bartosz Cieślik-Wolski, Katarzyna Wojciechowska-Durczyńska, Beata Smolarz

**Affiliations:** 1 Laboratory of Cancer Genetics, Department of Pathology, Polish Mother’s Memorial Hospital Research Institute, Rzgowska 281/289, 93-338 Lodz, Poland; 2 Clinical Department of Thoracic Surgery and Respiratory Rehabilitation, Nicolaus Copernicus Provincial Multispecialty Center of Oncology and Traumatology, Pabianicka 62, 93-513 Lodz, Poland; bw.cieslik-wolski@kopernik.lodz.pl; 3 Clinic of Endocrinology and Metabolic Diseases, Polish Mother’s Memorial Hospital Research Institute, Rzgowska 281/289, 93-338 Lodz, Poland

**Keywords:** microplastics, nanoplastics, lung cancer, environmental pollution, particulate matter

## Abstract

The ubiquitous environmental pollution with micro- and nano-sized plastic particles (MNPs) is a current and significant problem today. At the same time, lung cancer is responsible for the largest number of cancer-related deaths worldwide. Many research groups have investigated the relationship between lung cancer development and exposure to MNPs in recent years. Studies have demonstrated that these particles could enter the respiratory system in a variety of ways—both directly through inhaled air and through the bloodstream, and through internalization in the intestines and other digestive organs. Data regarding the possibility of their aggregation in the respiratory system, thyroid gland, and brain are also concerning, as the harmful effects of MNPs have been proven to depend on their concentration and exposure time. The primary response of cells to plastic particles is an increase in oxidative stress. This is generated both by the cell itself (especially macrophages) and induced by damage caused by mechanical damage to cellular organelles by MNPs. The consequences of MNP exposure can include metabolic disturbances, DNA damage, and mutations, ultimately inducing neoplastic transformation in healthy cells. This can lead to changes in tissue architecture and increase their susceptibility to other pathogens, such as pathogenic microorganisms or heavy metals. These, in turn, can be internalized along with MNPs, forming a corona surrounding them.

## Simple Summary

Non-small cell lung cancer, characterized by its high prevalence and mortality, has been associated with environmental pollution. One of the most common and relatively recently studied pollutants is microplastics. Numerous studies conducted worldwide have documented its presence in various environments and in human organs, predominantly in the lungs and intestines. Research exploring the impact of microplastics on human lungs using both in vivo and in vitro models has indicated a potential correlation with the incidence of lung cancer. This review summarizes the current state of knowledge regarding the impact of microplastics on non-small cell lung cancer, as well as methods for identifying and imaging microplastics in environmental and biological samples. This review summarizes the current state of knowledge regarding the impact of microplastics on non-small cell lung as well as methods for identifying and imaging microplastics in environmental and biological samples.

## 1. Methods

The literature review was conducted between 21 July and 15 September 2025. The databases of Science Direct, Google Scholar, and PubMed have been searched for keywords such as “microplastics, nanoplastics, lung cancer, non-small cell lung cancer, microplastic assessment, and NSCLC therapy” in the date range 2019–2026. The inclusion criteria include at least two of the keywords, published in the date range, or that describe an important molecular mechanism associated with microplastics. The preferred language of the publication was English.

The Science Direct example search string with keywords “Microplastic AND lung cancer” showed 1806 articles, of which 703 were research papers, and after reading titles and abstracts, only 2 of them matched the inclusion criteria. In the initial literature search, 130 papers from the aforementioned databases received full-text reads, and 76 of them were included in the review. Additional searches led to the inclusion of another 53 papers in the article. The number of cited papers by type and methodology in the topic of microplastics: 35 review papers, 10 papers describing research based on human samples, 16 papers based on mouse or rat models, 22 papers based on in vitro models, and 3 papers that used both models in the research. Additional cited papers describe environmental pollution and microplastic assessment techniques.

## 2. Epidemiology of Lung Cancer and the Issue of Microplastics

Lung cancer is the most prevalent malignancy worldwide and the leading cause of cancer-related death, with over 2 million deaths reported in 2021 [1]. Despite a decline in incidence and mortality in developed countries, increasing global air pollution—particularly in low- and middle-income countries—continues to contribute to high mortality from lung cancer and other respiratory diseases. Epidemiological studies indicate that women are particularly susceptible due to prolonged exposure in households using solid fuel stoves [2]. In recent years, highly developed countries have achieved reductions in mortality through investments in health care, early diagnosis, and environmental pollution control, although these improvements have not been observed uniformly across all social groups [3].

Lung cancer arises from the epithelial tissue lining the respiratory tract, and its histological classification depends on the primary tumour site. Based on cell morphology, two main categories are recognized: small cell lung cancer (SCLC) and non-small cell lung cancer (NSCLC). The latter is further divided into three major subtypes:

(1) Lung adenocarcinoma, which represents approximately 40% of cases and 55% of non-small cell lung cancers, arises from the bronchial epithelium and is typically classified as a peripheral lung cancer. It usually presents as pulmonary nodules; however, its course may be insidious because of the tendency for early metastasis.
(2) Squamous cell carcinoma, accounting for 30–40% of cases, also originates from the alveolar epithelium. Approximately two-thirds of cases are located centrally, while one-third are peripheral. This subtype generally develops slowly but is frequently associated with bronchial obstruction and obstructive pulmonary disease.
(3) Large-cell carcinoma—a relatively rare variant of lung cancer, accounting for approximately 9% of cases. It is strongly associated with smoking and other carcinogenic exposures, often occupational in nature, and occurs more frequently in men. This subtype is characterized by a low survival rate, a high propensity for chest wall invasion, and early metastasis [4].

The most common type of lung cancer is non-small cell lung cancer, accounting for over 80% of all cases. Small-cell lung cancer (SCLC), by contrast, represents approximately 14% of cases and is classified as a high-grade malignant neuroendocrine tumour associated with long-term cigarette smoking [5]. SCLC has a worse prognosis because it is frequently diagnosed at an advanced stage and develops resistance to therapies. The average 5-year survival rate for lung cancer patients is 10 to 20% [6].

The widespread use of plastics underpins the functioning of numerous industries and supports daily life applications, primarily due to their durability. However, this very property of polymers such as polyethylene (PE), polystyrene (PS), and polyethylene terephthalate (PET) also represents their greatest long-term environmental challenge. Of the more than 450 million tons of plastic produced globally each year, only about 10% is recycled, and projections suggest that by 2050, annual plastic waste entering the environment could exceed 30 million tons [7]. As plastics undergo chemical and mechanical degradation in the environment, they fragment into progressively smaller particles. These are categorized as microplastics, defined as particles smaller than 5 mm, and nanoplastics, defined as particles smaller than 1 µm. Research on microplastic pollution initially focused on marine ecosystems, where their adverse effects on aquatic organisms and freshwater environments were first documented [8]. Over the past decade, research has increasingly focused on the presence of microplastics in soil and even in the atmosphere. Their widespread occurrence reflects the pervasive influence of human activity and the complex interdependencies within the global environment. The removal of microplastics from freshwater systems remains highly challenging, and their neutralization in seawater is even more difficult, particularly given the capacity of plastic particles to be transported by the atmospheric processes. Aquatic pollution also facilitates the incorporation of plastics into food webs, where adverse effects have been documented across multiple trophic levels [9]. Micro- and nanoparticles of plastics, along with other pollutants such as heavy metals and volatile organic compounds, are a fraction of suspended particulate matter (PM) of various sizes, ranging from 2.5 µm to over 10 µm [10]. Smaller particles showed greater penetration capacity and, consequently, higher toxicity. PM with a size of 2.5 has been shown to directly damage DNA in lung tissue [11] and induce pro-inflammatory factors [12], thus inducing or promoting cancer progression. Clinical and experimental studies proved that PM-polluted urban areas were characterized by a significantly higher incidence of lung cancer in both non-smokers and smokers [13]. The importance of research addressing the increasing presence of microplastics in the environment and their potential impact on the respiratory system is increasingly emphasized. In the context of ongoing climate change and the expansion of global economic activity with its associated environmental impacts, lung diseases, including lung cancer, are likely to remain a major public health concern for many years to come [14].

## 3. Lung Cancer Diagnosis

Lung cancer is typically identified via radiography when fibrotic changes or nodular lesions, referred to as hyaline nodules, become visible in the lungs [15]. The radiographic appearance of lung cancer is highly variable. A suspected lung tumour on a conventional chest X-ray in anteroposterior and lateral views may be suggested by the presence of a round shadow or changes in the contour of the lung hilum. If a solitary, undefined nodule larger than 1 cm in diameter is present in the lung parenchyma, positron emission tomography combined with computed tomography may be helpful. This examination facilitates the differentiation between benign and malignant lesions, helps with further planned surgical treatment or radical radiation, and determines the indications for other tests or observation. Magnetic resonance imaging (MRI) is used to assess the tumour’s invasion of nearby structures such as the spine, mediastinum, chest wall, and diaphragm. These radiographic changes, however, do not necessarily indicate the presence of cancer. The clinical symptoms, such as cough, shortness of breath, chest pain, and hoarseness, also play a critical role in diagnosis. Because these symptoms are nonspecific, lung cancer is frequently diagnosed at an advanced stage [16]. More specific complications may also occur, such as upper limb pain, arm muscle atrophy, or bone damage, while brain metastases can manifest as headaches, vomiting, or even disturbances in vision [17]. The initial diagnosis is then confirmed with imaging using computed tomography and mucosal evaluation. The final step before treatment begins is to assess the genetic type of the tumour to guide treatment selection [16]. To facilitate earlier detection, recent research has focused on identifying molecular biomarkers of early non-small cell lung cancer (NSCLC) through liquid biopsy samples, a minimally invasive alternative to traditional tissue biopsies [18]. Specific microRNA panels seem to be promising in this context [19], as have exosomal long non-coding RNAs such as AL139294.1 or SOX2-OT, which may be utilized in screening tests [20]. Another innovation involves assessing the quantity and quality of platelets, with a focus on their modifications induced by the tumour and its microenvironment [18]. Methods combining radiological images and DNA methylation markers have also proven effective [21]. Additionally, artificial intelligence models based on neural networks are being developed to improve the accuracy and speed of lung cancer diagnosis using radiological images [22].

## 4. Lung Cancer Therapies

The treatment of diseases such as lung and other tissue cancer is dependent on the type and stage of the disease. Targeted metastasis control therapy and stereotactic body radiotherapy are among the most effective and commonly used treatment modalities, offering a lower risk of side effects and a higher likelihood of therapeutic response [23]. Consequently, combination therapies are frequently employed to maximize treatment efficacy. The breakthrough in lung cancer treatment was the discovery of the role of epithelial growth factor receptor (EGFR) and cytotoxic T cell antigen 4 (CTLA4) in the metabolic pathways of the NSCLC and further application of drugs based on tyrosine kinase inhibitors (TKI). The efficacy of TKI therapy relies on blocking EGFR signalling, thereby inhibiting cancer cell proliferation and division. However, TKI treatments are indicated only for NSCLC subtypes harbouring EGFR mutations or other driver mutations. In the absence of such mutations, treatment options typically include stereotactic or conventional radiotherapy [17].

A recent advancement in targeted therapy is the use of immune checkpoint inhibitors (ICIs), which enhance the immune system’s ability to combat cancer by blocking immunostatic proteins [24]. ICI targeting the Programmed Cell Death 1 and Programmed Cell Death Ligand 1 (PD-1/PD-L1) axis represents one of the most widely used and effective therapies in NSCLC [25]. This therapy is effective for NSCLC types with and without EGFR driver mutations. On the molecular level, the treatment relies on the PD-L1 on the cancer cell membrane binding with PD-1 on the macrophage lymphocytes T and B, thereby preventing the formation of an immunosuppressive tumour microenvironment mediated by suppressive cells such as tumour-associated macrophages (TAMs) and regulatory T lymphocytes (Tregs) [26]. The response to ICI monotherapy is limited, with rates ranging from 20 to 40%, and these therapies are generally administered sequentially following prior TKI treatment in advanced disease stages [25]. The DNA methylation status in cancer cells may help assess relapse risk and personalize treatment at an earlier stage, potentially improving therapeutic efficacy [27].

One of the most promising emerging therapeutic targets in lung cancer is cuproptosis, a recently identified form of copper-dependent cell death. The induction of this pathway involves copper ion overload within metabolic processes, leading to abnormal oligomerization of acylated mitochondrial proteins, including dihydrolipoyl transacetylase (DLAT), and destabilization of Fe-S cluster proteins. This cascade results in proteotoxic stress and disruption of cellular energy metabolism. Moreover, the various research conducted on lung cancer cell lines and clinical trials allowed us to link the dysregulated expression of cuproptosis-associated genes, such as genes coding ferredoxin 1 (FDX1), lipotransferase 1 (LIPT1), copper transporter 1 (CTR1), and ATPase copper-transporting α/β (ATP7A/B), to lung cancer progression. Experimental strategies aiming to selectively induce cuproptosis—such as targeting copper transporters modulating immune checkpoints via advanced drug delivery systems—may offer novel therapeutic options for lung cancer cells resistant to conventional therapies [28]. The potential of targeted delivery of Cu^2+^ ions with microplastics offers a potential treatment method, although there are no human studies yet [29]. Research by Zhang et al. (2025) highlights the potential of incorporating the disintegrin and metalloproteinase domain-containing protein 10 (ADAM10), known to interact with key signalling and adhesion molecules, in the diagnosis and treatment of lung cancer [30]. Treatment efficacy may be enhanced by increasing the chemosensitivity of cancer cells. One approach involves the use of S-adenosylmethionine (SAM), a methyl donor that can modulate autophagy and oxidative stress pathways. SAM has been shown to reduce the proliferation and viability of non-small cell lung cancer cells and to increase their sensitivity to chemotherapeutic agents [31].

The high incidence of NSCLC, coupled with the widespread occurrence of microplastics in inhaled air, naturally raises concerns about its potential role in the pathogenesis of this cancer and its clinical significance.

## 5. Global Burden of Microplastics and Perspectives on Its Reduction

Between 2010 and 2019, the average per capita plastic consumption in the EU was 112 kg/year, with packaging and agriculture as the leading sectors. While domestic production has declined, imports now account for most plastics. EU regulations have enabled nearly 40% recycling, although some plastic waste is exported to countries with less stringent standards [32,33]. For example, in Poland, less than 0.01 kg of plastic per capita is emitted into the oceans, and 0.37 kg is not recycled or is disposed of incorrectly, whereas in China, 0.049 kg of plastic reaches the oceans, and 8.56 kg is not recycled [34]. In recent years, 79% of all plastic produced has been discarded or buried in the natural environment. Attempts are being made to remove it, but current technologies rely primarily on filtration and are used primarily in wastewater and water treatment plants. Methods used include adsorption and filtration, chemical or physical interaction [35], and the use of organisms to absorb and digest them (marine fungi, zooplankton, and bacteria). Currently, these technologies have limitations: decomposition leads to further contamination, the microorganisms act slowly, and filtration does not reach the smallest particles [36]. While the presence of microplastics in the environment is well documented, the extent to which they are internalized by the human body remains debated. Estimates suggest that up to 5 g per week could theoretically reach human cells, but actual exposure appears to be substantially lower. Median daily intake has been estimated at 184 ng/person for both children and adults, corresponding to approximately 6.4 ng/day for children and 40.7 ng/day for adults, specifically for the accumulation of microplastics in the 1–10 μm size range [37]. The data presented by Eberhart et al. in the 2024 review suggest lower median values of daily intake for adults: 151.9 MP/kg of body mass and over 549 MP/kg of body mass for children. Data collected by Boccia suggest that outdoor MP concentrations are lower than indoor and that the abundance of certain sizes and shapes of polymer particles is dependent on humidity, wind, location, and barometric pressure [38]. This implies that not only are people working with polymers more likely to intake MP inside buildings, but the office and the other workers are too. Research shows that the method and quality of room ventilation and the presence of it are crucial for air quality and ambient microplastics exposure [39,40]. Recently, concerns have also arisen about patients’ exposure to microplastics during surgery or drug infusions. A recently published study highlights the widespread presence of nanoparticles of various types of synthetic polymers—PE, polypropylene (PP), and PS—in commercially available saline and glucose infusions, which may have potential negative effects on patients [41], because inflammation was observed after long-term contact of tissues with PP [42].

Urban areas may exhibit higher MNP concentrations compared to rural settings, potentially increasing NSCLC incidence in polluted regions. Increased risk of MP inhalation in indoor environments such as vehicles and buildings, and described toxic effects, provide additional arguments for environmental monitoring and care for the patients’ surroundings, especially those with prolonged hospitalization.

## 6. Methods for Detecting Plastic in the Environment and Organisms

Despite the development of numerous methods for assessing microplastics in the environment and tissues, basing research and conclusions solely on a single method is not recommended due to the known limitations of individual technologies and the occurrence of false positives and negatives. The most popular method and the one considered as the gold standard [43] is Fourier Transform Infrared Spectroscopy (FTIR). It is based on the measurement of the absorption or emission of the infrared spectrum by a sample and allows for the identification of functional groups present in the studied polymers. However, it enables imaging of particles above 20 µm and is highly susceptible to external factors (diffraction limitations). The micro-FTIR technique allows for the evaluation of individual spectral bands thanks to focal arrays, which in turn translates into greater efficiency [44]. Due to the mentioned particle size limitations, FTIR is frequently used with Raman spectroscopy (RS), based on Raman scattering radiation. RS is offering a wide range of qualitative sample analysis. The method’s drawbacks include the long time required to obtain results and the imaging of particles smaller than 20 µm. Resolution can be improved by analyzing the tested particles using flow technology [45] or reducing the procedure time by supporting the process with artificial intelligence algorithms [46]. Micro-Raman spectroscopy (MRS), on the other hand, allows focusing the laser on a narrow research area to evaluate micrometre-sized objects [47]. Further development of this technology is Stimulated Raman Spectroscopy (SRS), offering the ability to detect the nanoparticles in fresh human lung tissue and improved efficiency over MRS [48]. The method with a significantly faster time of measurement is the laser-assisted direct infrared (LDIR) imaging, which allows for rapid (counted in seconds) and comprehensive assessment of MNP particles as small as 10 µm in the environment. However, this technique has a narrower detection bandwidth than FTIR and Raman spectroscopy [49]. Another technique utilizing spectroscopy is optical photothermal infrared spectroscopy (O-PTIR), a Quantum Cascade Laser technique utilizing IR and Raman spectroscopy that allows for highly efficient qualitative assessment of particles by measuring infrared absorption during thermal expansions of the sample induced by an infrared laser. Careful sample preparation is necessary, as the measurement can be distorted by the presence of water vapour [50].

Another approach for MP assessment is the optical microscopic analysis using fluorescent dyes. It is a relatively inexpensive method, but it requires labour-intensive sample and particle preparation and offers relatively low accuracy due to particle size and the presence of contaminants in the sample. Hydrophobic dyes such as Nile red, Rhodamine B, Safranin T, and fluorescein are used, which significantly increase the visibility of the examined particles [51], but the aforementioned spectroscopy technologies can also be used with a microscope as a method of MP visualization. Another microscopy-based method is the energy-dispersive X-ray scanning electron microscope (SEM). It is one of the more complicated and expensive techniques with low throughput, but it provides the ability to assess the surface and elemental composition of specifically selected particles [52]. The newly developed support of deep learning algorithms overcomes the low throughput drawback, enabling a significant reduction in the time of classification of tested particles [53]. The combination of atomic force microscopy and infrared spectroscopy leads to the creation of the AFM-IR, a method offering a resolution at the atomic level thanks to a sharp probe sampling the surface of the sample and the ability to assess the sample’s chemical structure in the infrared spectrum. This method found application in in situ analysis of MPs in water and tissue samples. Due to specialized equipment and the level of accuracy this technique requires, experience is burdened with a high cost [54].

Mass spectrometry allows obtaining information on the structure and molecular weight of the tested material at the expense of a narrow evaluation range and problems with the quantitative assessment of the plastic in the sample [55]. Pyrolysis–gas chromatography–mass spectrometry (Py-GC-MS) combines mass spectrometry and chromatography of evaporated sample material. The limit of the very low mass of the assessed sample is a drawback, but the sample does not require prior preparation. Complete sample combustion allows for the identification of multiple types of polymers simultaneously with high accuracy [56,57]. The thermal decomposition of the sample is also used in thermogravimetric analysis of microplastics (TGA)—a method based on measuring the mass loss of a sample during heating at a controlled rate. Combined with other methods, such as mass spectrometry, it provides a broader analysis and proves useful for assessing plastic concentration and its chemical characteristics [58].

## 7. Microplastics in Organisms and Tissue—The Molecular Basis of Plastic Toxicity

A study by Liang et al. investigated how the toxicity of polystyrene nanoplastics toward the lungs varies in different biological fluids. The potential for particle aggregation and deposition was dependent on their functional groups and the ambient pH. Notably, in artificial lysosomal fluid, particle aggregation occurred more rapidly than in artificial lung fluid, regardless of the specific characteristics of the PS nanoparticles [59]. Analysis of the lower respiratory tract in humans revealed that the majority of contaminants were cellulose fibres and their derivatives, such as viscose, as well as cotton fibres. The highest accumulation of internalized nanoplastic particles was observed in the thyroid, kidneys, and brain, likely due to the structural characteristics of these organs [60]. The study of Zhu et al. documented that the highest concentrations of MP were in lung tissue, with over 14 particles per gram and varying in size from 20 µm to 100 µm, with PCV being the predominant type of polymer [61]. Cellular studies using Calu-3 epithelial cells and THP1 macrophages demonstrated differential responses: epithelial cells absorbed NPs but gradually excreted them, whereas THP1 macrophages retained the particles in the cytosol, leading to accelerated oxidative stress. Toxicity assessments further indicated that aminated microplastics exhibited higher cytotoxicity compared to carboxylated plastics or particles lacking functional groups [62]. The relationship between plastic composition and its accumulation sites in the human body is reflected by the differential distribution of various plastic types across organs. These data are summarized in Table 1. Particle size and morphology further modulate toxicity. Particles exceeding 20 µm are retained within macrophages and are not efficiently cleared from the respiratory tract via mucociliary transport. Moreover, particles with irregular or complex morphologies may serve as vectors for pathogens, as their structural niches can facilitate microbial colonization [63]. A size of approximately 20 µm is considered the upper limit for particles to penetrate organs and cross the blood–brain barrier [60,64]. The shape of microplastic particles is a critical determinant of their potential to mechanically damage cellular membranes. Experimental data indicate that exposure to ultraviolet (UV) radiation accelerates plastic degradation, producing smaller fragments with irregular and often sharp edges. Consequently, UV-aged microplastics are significantly more harmful to cells compared to their unweathered counterparts due to their enhanced ability to penetrate and disrupt membrane integrity [60]. Studies on aquatic and terrestrial organisms, as well as plants, confirm that microplastics exert similar toxic effects across all living systems. In onion (Allium cepa) cells, exposure to nanopolystyrene induced oxidative stress, resulting in DNA damage and reduced DNA repair activity. The observed cellular toxicity was both dose- and time-dependent, with concentrations of 100 μg/mL completely suppressing antioxidant defence mechanisms and triggering cell death [65,66].

### 7.1. Mechanisms of Microplastics-Induced Toxicity

Microplastics resemble asbestos fibres in terms of their cellular effects. Asbestos, once considered a revolutionary mineral material, is now discontinued due to its extreme durability, difficulties in safe disposal, and severe health hazards. Both plastics and asbestos have been detected in human lungs and surrounding tissues, indicating their ability to migrate within the body. Their cellular toxicity is largely linked to mechanical damage to organelles and the induction of oxidative stress. Asbestos exposure is a well-established cause of mesothelioma, a malignant tumour of the pleura. Plastics, however, are now far more widely used than asbestos ever was, especially since the industrial use of silica fibres has been restricted to essential applications [72]. Studies suggest that some hydrocarbon-based plastics may undergo partial degradation in cells—unlike mineral asbestos fibres. Yet, complete decomposition of plastic particles requires strong oxidants, which are absent in living organisms. Instead, oxidative stress conditions can modify these particles, but this process has been proven to contribute more to cellular damage than to the actual degradation of plastics [73]. The toxicity of MPs, however, results not from the concentration but from the duration of exposure caused by their accumulation in organisms. Macrophages are particularly susceptible to damage caused by MP accumulation, as they are among the first immune cells to conduct phagocytosis and respond to threats in organs exposed to external factors and pathogens. Following internalization, MPs can drive macrophage polarization or transformation into harmful phenotypes. Independent studies on both synthetic and natural materials (e.g., polylactic acid) have demonstrated that macrophages internalize diverse particle types and attempt to degrade them within lysosomes. However, lysosomes overloaded with MPs may become leaky, triggering inflammation, altering macrophage behaviour, and inducing the secretion of pro-inflammatory mediators such as interleukin-8 (IL-8). The observed effects vary according to particle characteristics: toxicity increases with smaller size, higher concentration, and prolonged exposure. Additional determinants, including particle shape, physicochemical properties, and the presence of a protein corona, further modulate these interactions. The corona plays a particularly important role by mediating binding with proteins and other biomolecules, facilitating interactions with surrounding organisms and compounds, and enhancing the likelihood of macrophage phagocytosis. This relationship was exemplified in a study by Cui et al., which demonstrated that plastics lacking amine and carboxyl residues were inert to mouse macrophages and did not induce adverse effects [74].

### 7.2. Microplastic-Induced Damage Cell Coping Mechanisms

One mechanism by which cells cope with oxidative stress induced by microplastic exposure is the secretion of extracellular vesicles (EVs) to eliminate excess toxic agents and restore redox homeostasis. However, these EVs can be taken up by neighbouring cells, propagating oxidative stress over a wider area. This pleiotropic response is predominantly observed in immune and epithelial cells; for example, when lung epithelial cells secrete EVs to the lung surface liquid. Studies have demonstrated that certain microRNAs (miR-17, miR-20a, miR-21, and miR-145), vascular endothelial growth factor (VEGF), and pro-inflammatory mediators such as IL-8 and tumour necrosis factor-alpha (TNF-α) are transported within these vesicles along with cytosolic components and free radicals. Consequently, uptake of these EVs can promote cellular behaviours associated with carcinogenesis, including increased proliferation and reduced susceptibility to apoptosis. Further investigations indicate that the cargo of EVs released under oxidative stress is non-random. By secreting EVs, lung epithelial cells modulate their communication with immune cells, suggesting a signalling function. This stress-induced EV signalling has also been observed in various other human cell types [75,76] and in other organisms [77,78]. EV content is dynamic and dependent on location, type of stress, and cell type. For example, exposure to oxidative agents such as 4-hydroxonenal increases the release of tissue factor-positive extracellular vesicles by endothelial cells and fibroblasts, but not by monocytes [79].

Nuclear damage can also trigger inflammatory responses, mediated by the cytosolic DNA-sensing cyclic GMP-AMP synthase—a stimulator of the interferon genes (cGAS–STING) pathway. Activation of this pathway initiates a cascade of innate immune responses that are normally effective against pathogens and bacterial infections. However, when stimulated by micro- or nanoplastic particles, cGAS–STING signalling can contribute to cytotoxicity [80].

Studies have also confirmed the impact of plastic particles on mitochondria. Exposure to polystyrene and polypropylene particles has been shown to induce mitochondrial membrane depolarization, impair respiratory chain complexes, and reduce ATP production [67]. These effects may result from disruption of the proton gradient and altered mitochondrial membrane potential [81] or the previously occurred DNA damage [82]. In the case of stress, the amount of mitochondrial DNA itself increases due to the increase in the number of these organelles in response to the increased energy demand [83]. One of the most frequently observed and significant forms of DNA damage is 8-oxoguanine (8-oxoG), which arises from the action of hydroxyl radicals. This lesion can cause errors during DNA replication and transcription, leading to mutations, as 8-oxoG preferentially pairs with adenine instead of cytosine [79]. Histone proteins also undergo oxidative modification. One of the most characteristic signs of oxidative DNA damage is the presence of γH2AX histones—phosphorylated histones formed in response to double-stranded DNA breaks. Studies in rats also reported disturbances in the metabolism of glucose, purines, pyrimidines, sphingolipids, amino sugars, and nucleotides. These observed changes are characteristic of the early stages of the transition from normal cellular metabolism to a cancerous phenotype [83].

Microplastics also impact the endoplasmic reticulum (ER). Under stress conditions, protein folding is disrupted, triggering the unfolded protein response (UPR). This is particularly important because reactive oxygen species (ROS) play a critical role in the formation of higher-order protein structures. ROS are generated by the ER during protein modification, such as the formation of disulphide bonds, via the Ero1–PDI (protein disulphide isomerase) pathway, as well as through vitamin K epoxide reductase (VKOR). Maintaining ER redox homeostasis requires continuous handling of these radicals by PDI proteins, imposing additional stress on antioxidant systems, including the glutathione/glutathione disulfide (GSH/GSSG) pathway [84]. The endoplasmic reticulum (ER) can undergo rupture, leading to the release of Ca^2+^ ions into the cytosol, which disrupts cellular redox balance [85]. Transcriptomic analyses further confirm that polystyrene microplastics induce ER stress through activation of the PERK/eIF2α/CHOP signalling pathway [79].

Exposure to very high or unusually elevated concentrations of microplastics can induce necrotic changes in macrophages. Multiple independent studies have demonstrated that polystyrene microplastics stimulate macrophages to secrete pro-inflammatory mediators, including interleukin-6 (IL-6), interleukin-1β (IL-1β), and TNF-α, as well as to activate Toll-like receptor (TLR) signalling pathways. The specific effector molecules secreted and the pathways activated vary depending on macrophage subtype and anatomical location. Additional material modifiers, such as functional groups and particle surface charge, also influence macrophage polarization and secretion, but the results remain inconsistent regarding the effects of specific modifications. Particle surface charge strongly influences protein corona formation, with cationic particles showing increased toxicity due to enhanced internalization driven by interactions with the negatively charged cell membrane [86,87]. Ferroptosis may also be induced through MP effects on mitochondria. Oxidative stress reduced the concentrations of glutathione peroxidase 4 (GPX4) and ferritin heavy chain 1, which is responsible for iron storage in the cell. Iron released from ferritin contributed to the generation of additional free radicals, inducing lipid peroxidation and cell death. The toxic effect of MP could be mitigated by the antioxidant N-acetylcysteine [26].

Changes induced by oxidative stress or mutations often promote the formation of an inflammatory microenvironment conducive to fibrosis and carcinogenesis [88]. In lung cancer, the most frequently activated pro-inflammatory molecules are TNFα and interleukins, which, in turn, through inflammatory signalling pathways such as NF-kB (nuclear factor kappa-light-chain-enhancer of activated B cells) and STAT3 (signal transducer and activator of transcription 3), influence a number of factors such as the cellular matrix, growth factors, and angiotensin-converting enzymes, or directly influence immune cells and tissue-building cells, e.g., fibroblasts or epithelial cells [89]. Those changes can lead to the creation of a fibrous niche rich in activated fibroblasts—a microenvironment often associated with elevated risk of tumour development [26].

Nitrogen compounds also pose a health risk, as they can be present in particulate matter (PM) originating from fuel combustion or industrial processes. Their reaction with atmospheric water leads to the formation of secondary pollutants such as nitric acid (HNO_3_) and ozone. These substances either generate or contribute to the formation of reactive oxygen and nitrogen species (RNS). Moreover, certain plastics exposed to UV radiation can release RNS and aminoxyl radicals. These highly reactive compounds interact with other molecules, leading to the formation of additional toxic derivatives, including peroxynitrite—a potent biological oxidant. This represents an additional mechanism contributing to cellular stress and the long-term toxicity of microplastics [74].

### 7.3. Role of the Microplastics Modification and Additives

Various additives, including bisphenols, functional chemicals, and plasticizers such as phthalates, are incorporated into plastics to provide flexibility and other functional properties. These compounds can leach into the environment under external conditions. Evidence indicates that the toxicity of bisphenols, phthalates, and persistent organic pollutants (POPs)—such as pesticides, flame retardants (e.g., PBDEs), and combustion by-products (e.g., dioxins and furans)—exceeds that of the plastic polymers themselves [90]. Studies on rats indicate that compounds such as phthalates can even lead to disorders of glucose metabolism and insulin secretion by pancreatic B cells [91]. Furthermore, plastics in the environment degrade due to mechanical friction and UV radiation, creating microplastics with very irregular, often sharp shapes and variable structures. Microplastics often used in production experiments are spherical and smooth, which likely influences their actual toxicity and their interaction with cells, and therefore the research results [92].

In the natural environment, these degraded microplastics can readily adsorb co-existing environmental pollutants, including heavy metals, organic compounds, and pathogens. It has been hypothesized that, rather than the microplastic particles themselves, metal ions accumulated on their surfaces and internalized by cells along with the microplastic may be primarily responsible for disrupting cellular redox homeostasis [93].

### 7.4. Biofilm on Microplastic Particles

Biofilm formation on MPs is particularly important for the adsorption of co-occurring pollutants, and research indicates that this biofilm also substantially enhances the potential of cellular internalization of MPs [90]. The composition of the biofilm on each particle depends on both the current and previous environmental conditions to which the particle has been exposed. Within organisms, the protein corona may acquire new signal proteins and functional groups as it traverses various organs—reflecting the highly dynamic and relatively labile nature of the biofilm [73]. Studies examining the significance of differences in the environmental impact of biofilm composition have shown that microplastics with freshwater biofilm are internalized through macropinocytosis and phagocytosis, while microplastics found in seawater are internalized primarily through macropinocytosis. Phagocytosis is initiated by specific ligands on the cell membrane surface, activated by factors on the phagocytosed particle. To date, microplastic particles have been documented to contain a variety of biomolecules, including proteins, humic and fulvic acids, amino acids, lipids, polysaccharides, and carbohydrates, which collectively alter the physicochemical properties of the particle surface. Notably, although proteins often constitute a smaller fraction of the corona, the protein components play a primary role in facilitating particle internalization by phagocytes and macrophages. Proteins derived from freshwater environments, in particular, were observed to significantly enhance phagocytosis. The composition and functional properties of proteins, rather than their abundance, are critical, as internalization is mediated by the activation of membrane receptors specific to particular ligands. Two primary factors have been associated with the increased internalization of micro- and nanoplastics by macrophages and other immune cells: the intrinsic ruffling of their cell membranes and enhanced electrostatic interactions between biofilm-coated MPs and the cell surface. Furthermore, various salts, including Ca^2+^ ions present in the MNP biofilm, facilitate pinocytosis. In addition to active uptake, nanoplastics can passively enter cells via pinocytosis due to their small size [94,95]. Zeta potential (ζ-potential) is a key physicochemical parameter of plastic particles, reflecting their surface charge in aqueous environments and influencing their behaviour in the water column. It governs aggregation, dispersion, and interactions with organisms. This parameter can be modified during production by incorporating additives designed to promote particle aggregation in water, facilitating their capture or filtration. While a positive zeta potential is generally associated with increased toxicity, some studies have reported higher toxicity for negatively charged particles [74]. Collectively, these factors—biofilm formation, protein corona composition, cellular uptake mechanisms, and particle surface properties—determine the behaviour, bioavailability, and toxic potential of micro- and nanoplastics in biological systems.

## 8. The Impact of Plastic on the Risk and Course of Lung Cancer

Due to the crucial role of the respiratory system in responding to particulate matter and microplastic contamination, respiratory disorders have received particular attention. It has been discovered that particles <5 µm can reach the alveoli with inhaled air. Larger fragments are removed by the cilia and mucus of the bronchi and lungs through sedimentation into the esophagus and then into the digestive system. The efficiency of this mechanism is dependent on the particle location in the lungs. For example, more MPs were detected in the left lung, but this may be due to its greater ventilation [73]. The presence of fibres larger than 5 µm was also observed, which were allowed to penetrate so far due to their specific shape [69]. Once in the tissue, these particles can induce the development of pulmonary edema by increasing the expression of the *TRPV4* gene, responsible for coding the TRPV4 osmosis regulation ion channel [94]. Analyses have shown that healthy lung epithelial cells, such as BEAS-2B, are more susceptible to the negative impact of microplastics than A549 lung cancer cells. This suggests that MPs promote the induction of cancer formation rather than its progression [96]. Notably, the highest concentrations of microplastics in the human body have been detected in the lungs and intestines, with respiratory exposure constituting a major route of entry. Smaller particles exhibit prolonged retention within the respiratory tract due to reduced efficiency of mucociliary clearance mechanisms associated with their size. For particles larger than 1 µm, their transport towards the esophagus was much more efficient [97]. The presence of a mechanism for removing plastic particles from the respiratory system to the digestive system is confirmed by the results of a study of the effect of PS nanoplastics on the liver of mice. Both inhalation and ingestion of NP resulted in damage to this organ, evidenced by the increased presence of polyunsaturated fatty acids, characteristic of oxidative damage [98]. Slower clearance of small particles from the respiratory tract is an additional risk factor, as smaller particles are characterized by higher internalization. Despite the greater susceptibility of healthy cells to MPs, more MPs were detected in tumour tissues, and their number increased with age. This may be due to greater epithelial permeability but also to less efficient ciliary transport, allowing more time for particles to be internalized by cells. Another important finding is the observation that fibres found in the lungs have a significantly rougher surface than fibres examined in air, which may be a manifestation of the chemical interaction of cells with MPs [15]. In line with these observations, A549 cells were found to be characterized by higher MP internalization and lower accuracy of mutual adhesion [99], and this may be the reason for the detection of higher levels of foreign particles in lung tumours. Another study suggests that plastic particles detected in the lungs may not originate exclusively from inhaled air. Evidence indicates that microplastics can be internalized by intestinal villi, transported via the bloodstream to pulmonary alveolar capillaries, and subsequently accumulate within these vessels due to their restricted luminal diameter [73]. Interestingly, polyacrylamide particles in the blood did not exhibit cytotoxicity or affect circulation. However, after 16 weeks, their degradation products were detected in the intestinal walls [100]. The data presented indicate that even short-term exposure to MNP may have long-term effects, as it is not possible to completely eliminate all aggregated particles from the lung tissue [101], and long-term inflammation promotes the development of cancers such as liver cancer or lung cancer [93]. The presented data suggest that utilizing microplastics as targeted drug delivery platforms is unlikely to be feasible [102].

## 9. The Impact of Plastic on Cellular Pathways in Lung Cancer

### 9.1. mP Impact on BEAS-2B Cells

The multifaceted adverse effects of MP on the respiratory system have been consistently reported in numerous publications and meta-analyses [103,104,105]. The initial step leading to cellular damage is the uptake of MP particles. In BEAS-2B lung epithelial cells, internalization occurs through an integrin-dependent endocytosis pathway, particularly via integrin α5β1, which plays a critical role in cell motility and mechanosensing. Increased expression of this integrin has been associated with enhanced particle uptake [106]. The toxicity of micro- and nanoplastic particles in lung cells develops gradually, resembling their effects in other cell types, and involves the induction of inflammation and activation of immune responses that ultimately contribute to tissue and cellular dysfunction [107]. Oxidative stress has emerged as a key driver of cellular dysfunction, initially characterized by increased concentrations of GSH and GSSG proteins, followed by disturbances in purine metabolism that disrupt cellular homeostasis and alter the NAD+/NADH ratio in BEAS-2B cells [108]. Further changes included disruption of DNA repair systems. BEAS-2B cells exposed to polystyrene nanoparticles showed reduced base excision repair capacity and increased activation of survival pathways, including AKT and ERK phosphorylation. These cells also showed increased anchorage-independent growth and activation of pathways responsible for migration and invasiveness [96]. The process of metabolic alteration appears to be long-lasting, as demonstrated by studies on BEAS-2B cells exposed to PET nanoparticles (PET-NP). After 15 weeks of exposure, significant changes were observed only in genes related to epithelial–mesenchymal transition (EMT) and oxidative stress, without evident phenotypic alterations. Pronounced effects on metabolism and cell health became detectable after 30 weeks, including substantial DNA damage and anchorless growth. Transcriptomic analysis revealed that alterations in gene expression occurred from the earliest hours of exposure, with nine oncogenes—(K-ras protein) *KRAS*, (HGF receptor) *MET*, and (receptor tyrosine kinase) *RET*, as well as (transforming growth factor beta 1) *TGFB1*, (neurofibromin 1) *NF1*, (phosphatidylinositol-4,5-bisphosphate 3-kinase catalytic subunit alpha) *PIK3CA*, (RB transcriptional corepressor 1) *RB1*, (Ras like without CAAX 1) *RIT1*, and (U2 small nuclear RNA auxiliary factor 1) *U2AF1*—identified as the most deregulated. Further analysis showed that between weeks 15 and 30, a total of 295 genes displayed consistently altered expression, emphasizing the progressive cellular changes induced by PET-NP exposure. These findings highlight the long-term biological impact of plastic nanoparticles and underscore the toxicological risks associated with their environmental persistence [109]. Polyvinyl chloride (PVC) also exhibited harmful effects on BEAS-2B cells. It induced a decrease in cell viability, primarily through the MAPK and TGF-B pathways. These two signalling pathways play a key role in proliferation, differentiation, and apoptosis in various cell lines. PVC also disrupted lipid metabolism, thus affecting the structure of cell membranes. Furthermore, increasing sphingomyelin levels also affected the stability of these membranes and the cellular signalling of apoptotic and proliferative pathways. Dysregulation of amino acid metabolism was also observed, as it may cause further disturbances in basal metabolism, leading to increased levels of ROS [110].

Micro- and nano-plastic particles have been detected in cigarettes available on the market [111], and the harmful effects of cigarette smoke, dust, and microplastics on the respiratory system of animals have been described. Cigarette smoke and MPs acting together demonstrated the expected increased toxic effects in the form of oxidative stress, genotoxicity, anchorage-independent growth, invasiveness, and decreased oxidative stress response on the BEAS-2B cells. Compensatory mechanisms were also observed, such as cyclosporin A-dependent detoxification. High levels of oxidative stress were manifested by high expression of its marker genes: *SLC7A11* (a cystine and glutamate transporter, often upregulated in cancer), NQO1 (an antioxidant that reduces reactive quinones and stabilizes p53), and HSPA1A (a heat shock protein that stabilizes protein folding and is involved in the degradation of abnormal proteins). The expression of repressor genes, such as lysyl oxidase and *FN1*—which interact with extracellular receptors—was reduced. Moreover, increased senescence of lung epithelial BEAS-2B cells was observed, with cigarette smoke inducing this effect more strongly than microplastics [112].

### 9.2. MP Impact on A549 and Calu 3 Carcinoma Cells

**Figure 1:**
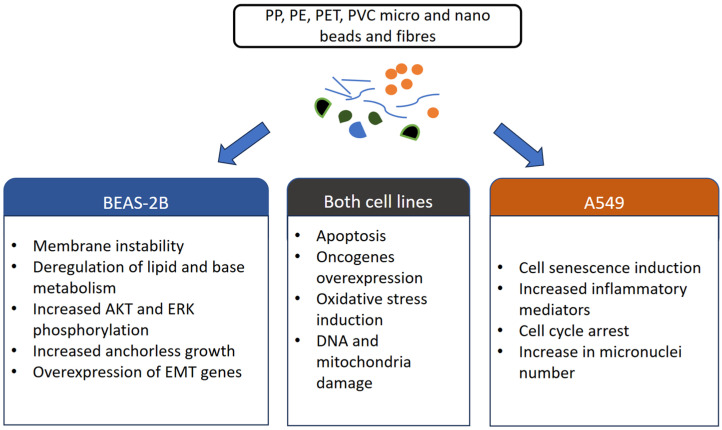
The impact of microplastics on BEAS-2B and A549 cell lines.

Individuals with NSCLC exposed to micro- and nanoplastics, as well as ozone, may exhibit a reduced response to immunotherapy and a shorter time to relapse [113]. Studies using the A549 NSCLC cell line have confirmed a significant impact of microplastics on cancer cells’ metabolism. Polystyrene particles are readily internalized by these cells, leading to elevated levels of inflammatory mediators such as IL-8, NF-κB, and TNF-α [67]. PS-NP exposure also induced increased expression of proapoptotic genes of death receptor 5 (DR5), caspase-3, caspase-8, caspase-9, and cytochrome c, and resulted in cell cycle arrest. The mitochondrial metabolism was also deregulated, which negatively affected the energy cell balance. The magnitude of these effects was dose-dependent, reflecting the level of microplastic exposure [114,115]. Another research also shows that the toxicity of PS-NP on A549 cells, apart from dose, depends also on the particle size, shape, and additional functional compounds. The smaller size and increased surface area were the characteristics correlated with increased ROS and toxicity. Six hours of airborne exposure to PS-NP in sizes of 80 nm and 2 μm also proved a strong connection with mitochondrial damage and its toxic effects, such as reduced viability of the cells. Moreover, treated cells showed an increased number of micronuclei (chromosomes not incorporated into the nucleus), which is a strong sign of genotoxicity. Interestingly, the 2 μm PS particles showed very limited cytotoxicity on A549 cells [116]. The research by Jin et al. proved that PCV induces A549 cell senescence by raising oxidative stress [117]. Biodegradable plastics, made from plant-based raw materials, are now becoming widely used as more ecological substitutes for PE and other materials used to make everyday items and food packaging [118]. Polylactic acid is one such material that, due to its favourable properties, has recently been widely adopted in 3D printing for both industrial and household applications, including the production of toys and everyday gadgets. However, a considerable amount of waste is generated during processing and use, and subsequent degradation may lead to the formation of microplastics—not only through mechanical fragmentation but also during incomplete biodegradation under composting conditions. The microplastics effects on A549 cell line and BEAS-2B are summarised in the Figure 1. The results of a 2024 study by Garcia-Rodriguez et al. indicate that PLA nanoparticles have measurable effects on the respiratory system. In Calu-3 bronchial epithelial cells, exposure to PLA nanoparticles resulted in reduced cell–cell junction formation and decreased mucus secretion. DNA damage was also observed, with more pronounced effects following long-term (1–2 week) exposure compared to short-term exposure, particularly at higher nanoparticle concentrations. Prolonged exposure further induced the secretion of cell repair proteins and structural remodelling. Importantly, the polymer’s structural features that enable its bioavailability to soil organisms—a key aspect of its biodegradability—also facilitated uptake and internalization by human epithelial cells in an air-liquid model [119]. On the contrary, PLA did not seem to affect A549 lung cancer cells in a negative way, although the potential metabolism changes remain incompletely understood [120]. This raises concern, given that this material is used in medical implants [121,122].

### 9.3. MP Impact on In Vivo Models

Studies on the synergistic effect of PS-NP and ozone on respiratory cells in mice also demonstrated a higher dose-dependent induction of inflammation than either alone. The inflammation was induced by changes in linoleic acid metabolism and ATP synthase (ATP)-binding cassette (ABC) transporters [123].

Another interesting issue is the impact of foreign synthetic particles, such as microplastics, on the microbiome of both the respiratory and digestive systems. A 2024 study found no specific impact of PET nanoplastics on this environment, other than inducing dysbiosis. It was also noted that changes in the gut microbiota could negatively impact the well-being of the respiratory system [124]. Another study demonstrated that polyethersulfone (PES)—a material commonly used in medical devices, electronics, and automotive components—can disrupt the intestinal microbiota, leading to liver damage, and induce lung injury through respiratory dysbiosis [125,126]. The close interaction between the gut microbiota and the immune system is illustrated by a 2023 study demonstrating that quantitative and qualitative alterations in bacterial species within the digestive tract may contribute to the development of pulmonary fibrosis [127].

### 9.4. MP Increases Probability of Asthma Occurrence

MP particles suspended in dust can have a carcinogenic effect and induce and exacerbate asthma symptoms in healthy individuals. Data suggest that women are more susceptible to developing asthma due to environmental factors than men, but this is not conclusive [128]. Air pollution is particularly dangerous for patients with chronic lung disease, as they are more likely to experience pro-cancer changes in their respiratory systems, such as inflammation and the induction of oncogene expression and elevated levels of CD24—a known carcinogenesis marker [129]. MPs induce oxidative stress, which in turn downregulates the expression of tight junction proteins such as junctional adhesion molecule (JAM) and occludin, as well as the pulmonary surfactant protein A (SP-A). These alterations increase epithelial permeability and contribute to lung dysfunction [130].

## 10. Conclusions and Potential Clinical Applications

With global economic development and increasing plastic production, the impact of microplastics on human health has become a major focus of research. The expansion of monitoring centres and advances in particle imaging have improved the detection and characterization of microplastics in the environment. The ubiquity of those particles in everyday activities and workspaces, both offices and factories, indicates that human exposure to microplastics is practically constant. Studies to date indicate that exposure to micro- and nanoplastics can induce oxidative stress, activate cellular repair pathways, and mechanically damage organelles, disrupting multiple metabolic processes. Both short- and long-term exposure have been linked to carcinogenic effects in lung epithelial cells and other tissues. The microplastics human exposure data are derived only from post-operation material of various diseases or post-mortem assessment, because clinical trials investigating MP absorption and metabolism in humans are fraught with ethical challenges, making them difficult to conduct. The molecular effects on the human organism are still mostly extrapolated from in vitro models or studies conducted on other species with physiological similarities to humans. The MP’s ability to reduce viability and induce apoptosis has potential clinical application in targeted cancer therapy, but to date, no such research has been conducted, and the potential long-term MP aggregation complications are a serious risk. Combining current microplastic assessment and visualization technologies enables the detection of particles across a wide size range, down to nanometre resolution. However, their low throughput and limited sample capacity pose challenges when spatial data are required, which can limit the research on MP distribution in vivo. Continued investigation into the metabolic and systemic effects of microplastics is essential, alongside global efforts to develop more sustainable materials and processing methods.

## Figures and Tables

**Table 1:** Selected types of materials and locations in the human body in which they were detected.

Material	Symbol	Application	Localisation of Aggregation in the Human Organism	Source
Polypropylene	PP	Containers, fibres, protective masks, car parts	Liver, lungs, testicles, breast milk	[67,68,69,70]
Low- and high-density polyethylene	LDPE HDPE	Plastic bags, bottles, disposable cutlery (LDPE), industrial containers, bottle caps (HDPE)	Lungs, brain, liver, kidneys, testicles	[42,68,69,70]
Polystyrene	PS	Building insulation, containers, and vessels	Brain, lungs, kidneys, heart, thyroid	[60,69]
Polylactic acid	PLA	3D printing, packaging, implants	Breast milk	[71]
Polyvinyl chloride	PCV	Construction, cables, packaging	Liver, lungs	[61,68,69]
Cellulose derivatives		Textiles	Respiratory tract	[60,69]
Polyethylene terephthalate (polyester)	PET	Bottles, textiles, photovoltaic cells	Kidneys	[60]
